# Contrast-Enhanced Dynamic MR Imaging of Uterine Fibroids as a Potential Predictor of Patient Eligibility for MR Guided Focused Ultrasound (MRgFUS) Treatment for Symptomatic Uterine Fibroids

**DOI:** 10.1155/2010/834275

**Published:** 2010-08-16

**Authors:** Sang-Wook Yoon, Chan Lee, Kyoung Ah Kim, Sang Heum Kim

**Affiliations:** ^1^Department of Diagnostic Radiology, CHA Bundang Medical Center, CHA University, 351, Yatap-dong, Bundang-gu, Sungnam-si, Gyunggi-do 463-712, Republic of Korea; ^2^Comprehensive Gynecologic Cancer Center, CHA Bundang Medical Center, CHA University, Gyunggi-do 463-712, Republic of Korea

## Abstract

Magnetic resonance-guided focused ultrasound surgery (MRgFUS) is a non-invasive treatment approach for symptomatic uterine fibroids. One imaging characteristic considered in selecting patients who may benefit from MRgFUS of their uterine fibroids is the signal intensity of the fibroid compared with surrounding myometrium on T2-weighted MR images. Previous reports suggest that hyper-intense fibroids are less amenable to MRgFUS compared with iso- or hypo-intense fibroids. In this case study, we utilized contrast-enhanced dynamic MR imaging to further characterize the vascularity of a hyper-intense fibroid. Based on the results of dynamic T1-weighted contrast-enhanced images, we assumed that the hyper-intense appearance resulted from high fluid content rather than high vascularity and predicted that the fibroid would respond to MRgFUS. The patient underwent the MRgFUS without complication and reported significant decrease in fibroid symptoms at 3 and 12 months post-treatment. This case suggests that pre-treatment dynamic contrast-enhanced imaging used in conjunction with T2-weighted imaging may improve the criteria for selecting uterine fibroids amenable to treatment with MRgFUS, potentially leading to improved patient outcomes.

## 1. Introduction

Uterine fibroids are the most common tumors of the female reproductive tract. Fibroids have been identified clinically in at least 25% of women [1], and pathological analyses suggest that the prevalence of fibroids may be as high as 77% [2]. Although most fibroids are asymptomatic, approximately 25% are associated with symptoms that can have a significant impact on patient's quality of life, including prolonged or excessive menstrual bleeding, pelvic pain or bulkiness, dyspareunia, increased urinary frequency, and infertility [3].

Several options, each with varying degrees of invasiveness, are available for treatment of symptomatic uterine fibroids. These include among others, hysterectomy, myomectomy (abdominal or laparoscopic), uterine artery embolization, MR-guided Focused Ultrasound (MRgFUS), and hormonal therapy [4], which also is sometimes provided as an adjuvant to other therapies. Each of the treatments has its own benefits and disadvantages. For example, the benefit of hysterectomy is that the removal of the uterus is 100% effective in alleviating fibroid-related symptoms [5]. However, the procedure is invasive, requires general or epidural anesthesia, and typically involves several weeks of postoperative recovery time during which patients may be limited from engaging in daily activities, including work.

MRgFUS is a non-invasive treatment for uterine fibroids. It utilizes precisely focused ultrasound waves to generate and maintain high temperatures within the targeted fibroid, resulting in protein denaturation and coagulative necrosis [3]. MRgFUS is integrated with magnetic resonance imaging visualization to plan and guide treatment and to monitor treatment outcomes in real time. This allows precise thermal ablation of the treated fibroid while preserving surrounding normal structures.

While clinical studies demonstrate that MRgFUS is a safe and effective treatment for symptomatic uterine fibroids [6–8], not all patients are considered to be equally suitable candidates for the procedure [9]. Potential candidates are screened with pelvic MRI to determine if they meet patient selection guidelines. Factors considered for MRgFUS patient selection include the imaging characteristics and location of the fibroid within the pelvis, the number and size of fibroids, presence of structures obstructing the energy beam pathway, and the vicinity of the fibroid relative to vulnerable structures [10].

One imaging characteristic considered in the patient selection process is the fibroid's capacity to absorb heat, as the effects of MRgFUS result from the thermal ablation of the fibroid tissue. This is often assessed based on the signal intensity of the fibroid compared with the surrounding myometrium on T2-weighted MR images (T2WIs). Previous reports suggest that the average nonperfused volume (NPV), as measured by posttreatment contrast-enhanced image, obtained with MRgFUS in the treatment of hyper-intense fibroids, is lower than that achieved in iso- or hypointense fibroids [9, 11]. 

The following case report demonstrates successful treatment of a fibroid that is hyper-intense on T2WIs and suggests that contrast-enhanced dynamic MR imaging may be used to select a subpopulation of uterine fibroid patients suitable for MRgFUS despite hyper-intense results on T2WIs.

## 2. Case Report

**Figure 1:**
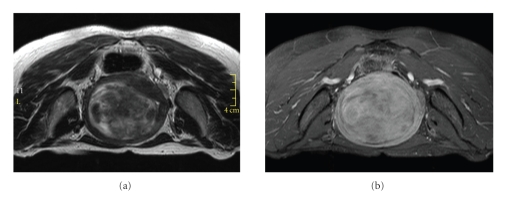
Patient screening MR images. (a) Axial T2-weighted image showing a heterogeneous fibroid, hyper intense relative to the uterine wall. (b) Contrast enhanced Axial T1-weighted screening MR image, showing enhancement of the fibroid.

A 47-year old premenopausal Asian female, with BMI of 20, was referred to our clinic by her gynecologist due to severe menorrhagia. Her Symptom Severity Score (on the 0 to 100 Scale of the UFS-QoL questionnaire [12]) was 56 points. This scale refers to patients with 21 points or more as symptomatic patients. The patient was referred for MRI screening to evaluate her suitability for the MRgFUS treatment. MRI showed a single intramural fibroid of 560 cc. Intensity of the fibroid relative to the uterine wall was hyperintense on T2WIs, and its texture was very heterogeneous (Figure 1).

**Figure 2:**
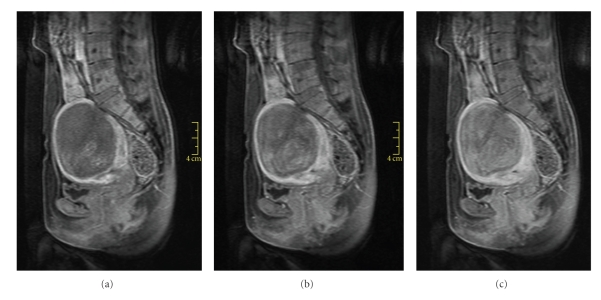
Contrast-enhanced sagittal T1-weighted screening MR dynamic images acquired at 30, 60, and 90 seconds post contrast injection (from left to right). Fibroid shows lower enhancement than the myometrium in the first 2 minutes postinjection.

To evaluate the fibroid's viability more fully, and as part of our screening routine for MRgFUS, T1-weighted MR images (T1WIs) with contrast were acquired. The contrast agent used was Optimark (Covidien Imaging Solutions, Missouri, U.S.). Dynamic T1-weighted gradient echo sagittal images were acquired every 15 seconds from the moment of injection until 180 seconds after the injection (TR 4.81 ms, TE 2.3 ms, matrix 256×115, thickness 3 mm, spacing 0.6 mm, FOV 30 cm) (Figure 2). T1-weighted axial fat-saturation images were taken 240 seconds after the injection (TR 594 ms, TE 11 ms, matrix 256×144, thickness 5 mm, spacing 1 mm, FOV 30 cm) (Figure 1). The images show low enhancement in the fibroid during the 120 seconds after the injection, despite high enhancement on the 240-second delayed scan. Based on the delayed enhancement, we assumed that the hyper-intense appearance of this fibroid resulted from the high fluid content rather than the high vascularity, and we decided to recommend MRgFUS to the patient.

**Figure 3:**
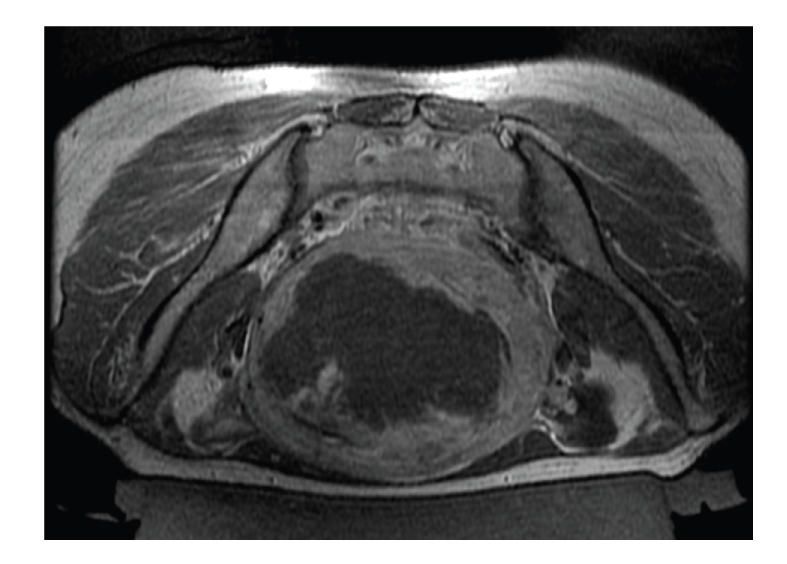
Contrast-enhanced axial T1-weighted MR image after treatment, showing nonperfused volume of 60%.

After consulting with the patient about the potential benefits and risks involved in treating her specific fibroid, she chose to undergo MRgFUS, knowing that results might be less than satisfactory due to the hyper-intense appearance of her fibroid. Treatment was performed using the ExAblate 2000 system (InSightec, Haifa, Israel) and SIGNA HDx MRI (GE Healthcare, Milwaukee, U.S.). T2WIs were acquired for treatment planning. The procedure required 128 focal ablations (sonications) with average energy of 3300 Joules. Treatment duration was 3.5 hours from first to last sonication. Average temperature achieved was 75°C. Contrast-enhanced T1WIs were acquired posttreatment, showing that 335 cc (60%) of the fibroid was nonenhancing (Figure 3).

The treatment was concluded without complications. During her followup phone call the next day, she reported returning to work and feeling well with no adverse events. The patient provided a similar report in a phone call 7 days posttreatment. At the 3-month followup, the patient's symptoms significantly improved, and her SSS score was reduced to 28 points. At that time, the patient returned for MR imaging, which showed 38% fibroid shrinkage. At 1-year followup, which included a clinical visit to evaluate patient's symptoms, her SSS was reduced to 13 points, which is considered as nonsymptomatic.

## 3. Discussion

In MRgFUS for symptomatic uterine fibroid, the non-perfused volume (NPV) of uterine fibroids has been found to correlate with treatment-related symptom relief, and to be inversely related with the need for repeated treatment (either MRgFUS or other therapeutic modalities) [8]. The success and durability of MRgFUS in relieving symptoms of uterine fibroids depend on appropriate selection of those patients in whom higher posttreatment NPV can be obtained. Therefore, patient eligibility is an important factor in achieving high NPV and significant symptom relief [10].

Based on previous experience, MRgFUS treatment of hyperintense fibroids usually results in relatively poor NPVs, and subsequent treatment failures [3]. The hyper-intensity of these fibroids on T2WIs is assumed to result either from high concentrations of fluids or from high vascularity. We assume that either scenario makes it difficult to achieve temperatures sufficient for thermal ablation of fibroid tissue, which are critical to the efficacy of MRgFUS. In case of high concentrations of fluids, the energy absorption by the tissue is low, resulting in low temperatures in the focus. In case of high vascularity the blood flow disperses the heat from the treated region. Consequently, the potential for low NPV on post-treatment imaging, subsequent insufficiency of symptom relief, and overall treatment failure in this patient were considered.

This case report shows that hyper-intense fibroids can be successfully treated with MRgFUS, however, the fibroids should be subcategorized based on their potential for successful treatment. T2WIs contribute to important patient-selection information regarding the location and structure of the fibroid(s) and coexisting pathologies. T1WIs with contrast examine the viability of the fibroid and, when performed dynamically, provide additional information on the rate of fibroid enhancement. Delayed enhancement may indicate that the fibroid has low vascularity and will absorb sufficient ultrasound energy to result in successful MRgFUS, even if the fibroid appears hyper-intense on the T2WIs.

We suggest that dynamic contrast enhancement imaging, in addition to fibroid intensity in T2WI, may provide important information whether or not a fibroid is suitable for MRgFUS. Although in this case report, other factors could have contributed to the treatment success, we assume that the dynamic contrast imaging during screening helped in patient selection. Additional studies are needed to verify this theory and establish a correlation between the pretreatment dynamic contrast enhancement imaging characteristics of a given fibroid and patient outcomes following MRgFUS.
